# Confusion in the Genesis of Art and Disease: Charles Laval, Paul Gauguin, and Tuberculosis

**DOI:** 10.3201/eid2603.AC2603

**Published:** 2020-03

**Authors:** Terence Chorba, John Jereb

**Affiliations:** Centers for Disease Control and Prevention, Atlanta, Georgia, USA

**Keywords:** art science connection, emerging infectious diseases, art and medicine, art and disease, about the cover, Confusion in the Genesis of Art and Disease: Charles Laval, Paul Gauguin, and Tuberculosis, Charles Laval, Paul Gauguin, Self Portrait, public health, tuberculosis, tuberculosis and other mycobacteria, bacteria, respiratory infections, history

**Figure:**
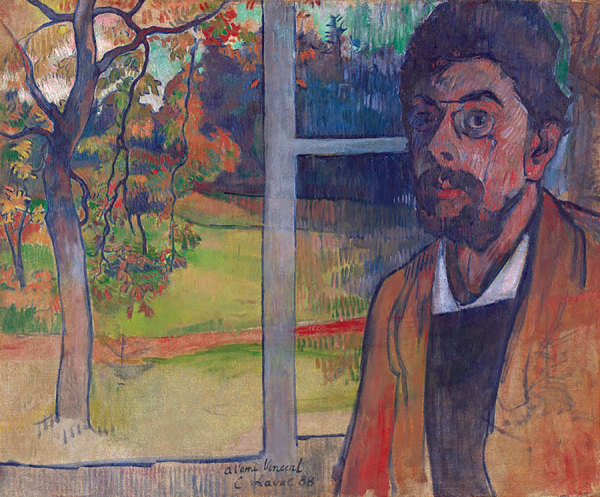
**Charles Laval (1861–1894), Self-Portrait, Pont-Aven, 1888.** Oil on canvas, 20 in x 23.8 in/50.7 cm × 60.4 cm. Van Gogh Museum, Amsterdam, Vincent van Gogh Foundation.

Charles Laval (1861–1894) was a Parisian painter whose brief life ended in an untimely death from underlying tuberculosis. He was a colleague and contemporary of two other post-Impressionists: Vincent van Gogh (1853–1890) and Paul Gauguin (1848–1903). In part because of misattribution, Laval’s work has receded into a historical footnote to the career of Gauguin, whose output far exceeded that of Laval. Gauguin, among the most renowned of the late 19th century European artists, was a pioneer of the Synthetist school of painting. This post-Impressionist movement encouraged artists to depict their emotions about the subject, beyond its outward appearance, with bold displays of color, relaxation of exactitude, and portrayal of form based on memory.

Laval studied under realist painter Léon Bonnat at the École des Beaux-Arts in Paris. In the summer of 1886, he became acquainted with van Gogh and Gauguin at Pont-Aven, a growing artist colony in Brittany. In April 1887, Laval and Gauguin set sail for Panama, an adventure that was ostensibly pursued for employment at the invitation of Gauguin’s brother-in-law, a Chilean with business interests in Panama. In June 1887, after several misadventures, Laval and Gauguin left Panama for Martinique, where they painted together before returning to France in November of the same year.

The emergence of Laval’s eccentric, flamboyant style coincided with Gauguin’s. The astonishing similarity of their works from that period has led art historians to conclude that a few of Laval’s paintings were incorrectly attributed to Gauguin, whose signature was added later to inflate market value. Femmes et Chevre dans le Village *(Women and Goat in the Village), presented on the next page (*Figure*), was undertaken in Martinique and bears a Gauguin signature but has been attributed by some to Laval.* The ascendance of Gauguin’s legacy and the neglect of Laval’s, in the context of their matching styles, could tempt us to surmise that Laval imitated Gauguin, but their paintings and their life stories leave open the possibility that their visions converged as they influenced each other. Only Laval’s early death deprived him, and us, of his potential for greater recognition.

During the brief sojourn in Martinique, Gauguin wrote to a painter friend back in Paris, Émile Schuffenecker, that he would be returning with a dozen of his own works, presumably to sell. However, in the various editions of the definitive Gauguin catalogue raisonné, a total of 18 works has been attributed to him from Martinique. The leading explanation for the surfeit of Gauguin-attributed works relative to the number that he reported to Schuffenecker is that some of these works may be those of Laval or other painters. Only 6 original remaining paintings are signed by and attributed to Laval, and all these date from after their time in Martinique.

Laval and Gauguin both returned to the school at Pont-Aven, but the friendship dissolved when Gauguin fell in love with Madeleine Bernard, sister of Émile Bernard, another Synthetist painter and van Gogh’s best friend, but Laval became engaged to her. Soon after the return to Pont-Aven, Laval developed tuberculosis. In 1890, he set sail for Cairo in search of a more favorable climate but returned to Paris and succumbed to an acute illness in 1894 at age 33. Tuberculosis patients easily fell prey to other illnesses because of their impaired pulmonary function and weakened constitution. Influenza was notoriously lethal for them. Madeleine also acquired tuberculosis and died in 1895. In our era of curative therapy, we forget the chronic natural course of tuberculosis: in the preantibiotic era, a third of its victims would survive longer than 5 years, perpetuating the epidemic and the dread that it evoked.

An infamously capricious agent of death, tuberculosis has taken away a great many artists prematurely. Through their creations, they could mourn the burden of mortality or conceal it defiantly. Laval seems to have traveled both pathways: his *Self Portrait,* inspired in an exchange with van Gogh in 1888 and praised by Gauguin, is featured on this month’s cover. In it, Laval gave us a brilliant exterior window view alive with splashes of color; he relegated his own face to one side, hauntingly somber, gaunt, peering out from cyan shadows wistfully. Does the gnarled tree reflect how Laval imagined himself, beaten down by tuberculosis?

In an 1895 letter, Gauguin mused that “in art, there are only two types of people: revolutionaries and plagiarists.” The resemblance of Laval’s work to Gauguin’s brought criticism to Laval. How fitting that tuberculosis, the source of Laval’s demise, is sometimes called “the great imitator” when clinicians are challenged by differentiating it from other diseases. Pulmonary tuberculosis and lung cancer commonly mimic each other, indistinguishable radiographically, sharing symptoms of cough, fevers, chills, loss of weight, and even similar presentations of metastasis. Similarly, intestinal tuberculosis mimics chronic inflammatory bowel disease, including Crohn’s disease and ulcerative colitis.

Although Gauguin’s most widely discussed, reproduced, and exhibited paintings were undertaken in Tahiti over a 10-year period toward the end of his life, the greater body of his work spanned a 35-year career. In contrast, Laval painted for barely a decade before tuberculosis halted the trajectory of his career. Had he fared better in his fight with tuberculosis, his artistic style could have matured independently, and his renown might have rivaled that of Gauguin. Had that happened, differentiating the work of these 2 artists would be easier.

**Figure 2:**
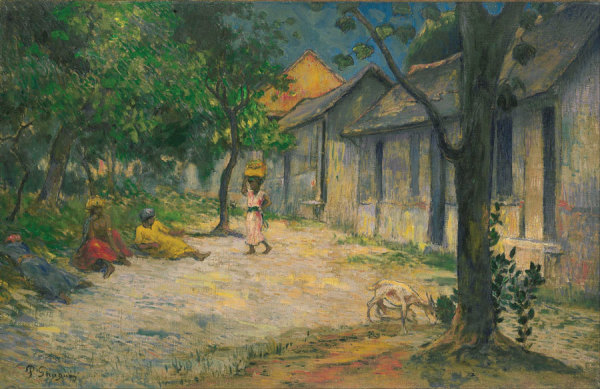
**Paul Gauguin (1848–1903), *Women and Goat in the Village* (*Femmes et Chevre dans le Village)*, 1887. **Oil on canvas, 45.7 x 71 cm. The Israel Museum, Jerusalem, America-Israel Cultural Foundation.
